# 

**Published:** 1855-02

**Authors:** 


VOL. IV. NEW SERIES. No. 2.

### THE NORTH-WESTERN MEDICAL AND SURGICAL JOURNAL:

PUBLISHED MONTHLY,

AT TWO DOLLARS PER ANNUM IN ADVANCE.

EDITED BY

N. S. DAVIS, M.D.,

PROFESSOR OF PATHOLOGY, PRACTICE OF MEDICINE AND CLINICAL MEDICINE.

AND

H. A. JOHNSON, A.M., M.D.

LATE LECTURER ON PHYSIOLOGY AND HISTOLOGY IN RUSH MEDICAL COLLEGE.

FEBRUARY, 1855.

CHICAGO:

J. F. BALLANTYNE, BOOK AND JOB PRINTER,

NEW POST-OFFICE BUILDINGS, COR. OF CLARK AND RANDOLPH-STS.,

OPPOSITE THE SHERMAN HOUSE.

VOL. XII. WHOLE SERIES. NO. 2.

